# A New Rehabilitative Mechanism in Primary Motor Cortex After Peripheral Trauma

**DOI:** 10.3389/fneur.2020.00125

**Published:** 2020-02-27

**Authors:** Florian Ph. S. Fischmeister, Ahmad Amini, Eva Matt, Raphael Reinecke, Robert Schmidhammer, Roland Beisteiner

**Affiliations:** ^1^Study Group Clinical fMRI, Department of Neurology, Medical University of Vienna, Vienna, Austria; ^2^Highfield MR Centre, Medical University of Vienna, Vienna, Austria; ^3^Department of Psychology, University of Graz, Graz, Austria; ^4^Ludwig Boltzmann Institute for Experimental and Clinical Traumatology, Vienna, Austria

**Keywords:** neuronal plasticity, functional connectivity, brachial plexus lesions, peripheral nerve reconstruction, functional magnetic resonance imaging (fMRI), motor cortex, rehabilitation

## Abstract

Homuncular organization, i.e., the neuronal representation of the human body within the primary motor cortex, is one of the most fundamental principles of the human brain. Despite this, in rare peripheral nerve surgery patients, the transformation of a monofunctional (diaphragm activation) into a bifunctional motor area (diaphragm and arm activation is controlled by the same cortical area) has previously been demonstrated. The mechanisms behind this transformation are not fully known. To investigate this transformation of a monofunctional area we investigate functional connectivity changes in a unique and highly instructive pathophysiological patient model. These patients suffer from complete brachial plexus avulsion with arm paralysis and had been treated with reconnection of the end of the musculocutaneous nerve to the side of a fully functional phrenic nerve to regain function. Task-based functional connectivity between the arm representations and the diaphragm (phrenic nerve) representations were examined in six patients and 12 aged matched healthy controls at ultra-high field MRI while they either performed or tried isolated elbow flexion or conducted forced abdominal inspiration. Functional connectivity values are considerably increased between the diseased arm and the bilateral diaphragm areas while trying strong muscle tension in the diseased arm as compared to the healthy arm. This effect was not found as compared to the healthy arm in the patient group. This connectivity was stronger between ipsilateral than between corresponding contralateral brain regions. No corresponding differences were found in healthy subjects. Our data suggests that the increased functional connectivity between the deprived arm area and the diaphragm area drives biceps muscle function. From this findings we infer that this new rehabilitative mechanism in the primary motor cortex may establish new intrahemispheric connections within the brain and the motor cortex in particular to reroute the output of a completely denervated motor area. This study extend current knowledge about neuroplasticity within the motor cortex.

## Introduction

The full functionality of the upper limb is one of the most significant motor function for humans. Common to all disruptions of arm functions regardless if they occur centrally or within the peripheral nervous system, a loss of arm function always has dramatic consequences. Thus, a comprehensive understanding of possible neuroplastic mechanisms allowing for the restoration of limb function is essential. The impressive power of cortical reorganization following brain damage has widely been proven; for a recent review of mechanisms within the somatosensory network see Harding-Forrester and Feldman (1); for the motor network see Beisteiner and Matt (2). While in these cases the typical stimulus for restructuring within the brain is the lesion of the brain, neuronal plasticity following peripheral damage occurs within a healthy brain. Here, only few neuroimaging studies exist reporting cortical reorganization in response to injuries of the peripheral nervous system. Most of these few studies show plasticity in non-primary brain areas, however, neuronal plasticity can also involve primary somatosensory and motor areas. This is of particular interest since the motor cortex is one of the most highly specialized cortices, allowing even the separation of single finger movements within the fMRI (3). Thus, studies within this domain are scarce and mainly concentrate on functional changes contra- and ipsilateral to the injured limb (4–6) or changes related to spinal cord injuries (7).

Possible mechanisms for neuroplastic changes following peripheral damage are not well-understood and range from loss of function to new specialization within the affected brain area (8, 9). Taking the perspective of primary motor cortex change in the information flow from the somatic periphery to the brain may lead to four distinct types of cortical reorganization: (1) no change in function, e.g., following reconnection of a transected median nerve (10, 11), (2) no change in function but new effector, e.g., after heterotopic hand replantation (12), (3) functional change, e.g., by optimizing a new motor function (13), and (4) loss of function, e.g., after amputation (14).

Recently, first evidence has been provided that neuroplasticity in primary motor cortex may go even beyond that. When connecting the ending of a differentiated musculocutaneous nerve to the side of an intact phrenic nerve, the task for the locally specialized primary motor cortex does not change, but a new task has to be added. Now, the cortical diaphragm representation has to perpetuate control of breathing but add independent control of arm flexion (15). Thereby a new bifunctional motor area is established. Interestingly, this goes along with a persisting activity of the former primary arm area (despite being completely disconnected).

Currently, it is not exactly known how this functional reorganization is realized, however, a recent study by our group indicates the establishment of a driving input of the denervated arm area to the diaphragm area which is now responsible for arm movements (16).

We here investigate which functional connectivity changes underlie this transformation of a monofunctional into a bifunctional primary motor area after peripheral trauma. Following our recent study we hypothesize that the denervated but highly active arm area establishes some kind of new functional connection to the diaphragm areas which are exclusively responsible for the generation of arm movements.

## Methods

### Participants

**Figure 1:**
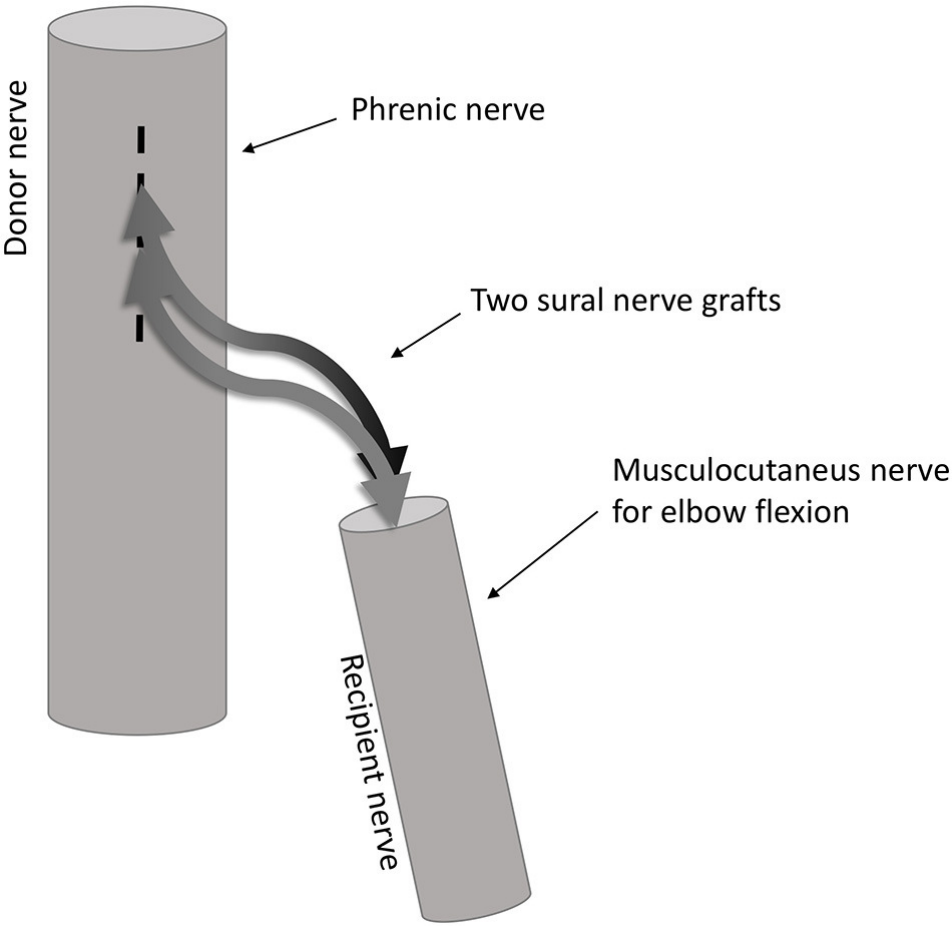
Illustration of the end-to-side coaptation nerve repair. The nerve fiber transfer from the phrenic nerve to the musculocutaneous nerve is done using two sural nerve grafts coapted end-to-side to the phrenic nerve and end-to-end to the musculocutaneous nerve.

Currently, about 20–30 patients with this new end-to-side reconstruction described below exist worldwide. In our study, six right-handed patients (26–47 years, median 37, and interquartile range 32.75–41, one female) could be included. All suffered from a complete traumatic brachial plexus avulsion (root avulsions and/or fiber disruptions) with arm paralysis. Since only complete lesion of the bracial plexus qualified for this type of intervention, avulsion of the plexus brachialis was initially defined via a thorough clinical investigation including structural imaging and clinical neurophysiology by an experienced clinical team and finally verified by the surgeon. All patients included in this study received an end-to-side coaptation of the distal stump of the musculocutaneous nerve to the side of the ipsilateral phrenic nerve using two sural nerve grafts (see Table 1 for further details and Figure 1 for a schematic illustration). Every patient followed a standardized physiotherapeutic and muscle stimulation protocol after surgery. Typically first arm movements do not start before 1.5 years after surgery. Clinical status documentation at the time of fMRI included chest radiography, thorax fluoroscopy, clinical video analysis of arm movement/breathing behavior and electromyography (EMG) to prove independent innervation of diaphragm and biceps muscles (15). With all patients, the post-surgical rehabilitation status at the time of the fMRI investigation showed a clinical muscle strength grading of 0–2 from 5, indicating that elbow flexion was not possible against gravity. None of the patients had any history of neurological or psychiatric illness besides the brachial plexus lesion. Since a direct comparison between end-to-side operated patients to those treated with an end-to-end technique can be found in Beisteiner et al. (15) we recruited twelve right-handed age-matched healthy controls (25–44 years, median 34.5, and interquartile range 29.5–40.25, one female) from the general population.

**Table 1:** Detailed characterization of the phrenic nerve patients.

**Patients**	**Age range**	**Injured arm**	**Accident date**	**Operation date**	**Scanning date**
1	25–30	Left arm	June 2010	February 2012	February 2013
2	30–35	Right arm	August 2011	February 2012	March 2013
3	35–40	Left arm	May 2009	April 2011	February 2012
4	35–40	Right arm	June 2010	December 2010	March 2014
5	35–40	Left arm	September 2010	February 2011	May 2013
6	40–47	Right arm	March 2012	February 2013	December 2013

All procedures performed in studies were in accordance with and approved by the Ethics Committee of the Medical University Vienna in accordance with the Helsinki Declaration of 1975 and its later amendments. Informed consent was obtained from all individual participants prior to participation in the study.

### Experimental Design

fMRI tasks were isolated tonic elbow flexion of (1) diseased/left or (2) healthy/right arm of patients/controls. The instruction was to flex the forearm slightly and generate a strong muscle tension without change of breathing. This task has repeatedly shown to generate reliable brain activation within related motor brain areas even if it was only imagined [c.f. for example Lotze and Halsband (17)]. Elbow flexion was trained and successful performance documented with clinical EMG outside of the scanner. Task (3) involved forced abdominal respiration with arms relaxed. Per run, three ON and four OFF blocks (rest with visual fixation) were applied (block duration 20 s). During ON phases muscle contraction was cycled four times with 2,500 ms tension followed by 2,500 rest (visual commands). Performance was monitored. Eight runs forced abdominal respiration, eight runs elbow flexion diseased/left arm, and four runs elbow flexion healthy/right arm were performed in a pseudo-randomized order. To allow for a high level of control analyses the experimental design included the possibility to (1) compare the patients' diseased arm with their healthy arm, (2) to compare the patients' diseased arm with the controls' arms, and (3) to compare the patients' healthy arm with the controls' arms.

### Statistical Analysis

#### Data Acquisition

Data for this study were acquired with a 7T Siemens MRI system (Siemens, Erlangen, Germany). fMRI activations in diaphragm areas are typically weak and 7T provides a unique functional contrast to noise situation and therefore optimizes the possibilities for reliable functional connectivity inferences (18). 7T data were recorded with a 32-channel phased array head coil, single-shot gradient-echo EPI, FOV of 230, TE/TR of 22/2,500 ms, matrix size of 128 × 128 and 39 interleaved axial slices with 3 mm gap, resulting in isocubic voxels of 1.8 mm edge length. High-resolution T1 anatomy was also acquired (0.7 × 0.7 × 0.7 mm). Head motion restriction was used and runs with insufficient compliance or large body movements were repeated (19).

#### Head Motion Analysis

Subject motion in particular in functional connectivity MRI pose a general problem in the analysis of fMRI data and thus has to be taken care of by compensatory spatial registration or regressing out motion estimates (20). To rule out systematic differences in motion between the patient and the control group we computed the framewise displacement metric as described previously (20, 21). This metric is defined as a six-dimensional time-series composed of the sum of the absolute derivatives of the realignment parameters. Rotational displacements are converted from degrees to millimeters by calculating displacement on the surface of a sphere of radius 50 mm. From this metric, we then compared the mean and maximum values per subject between the two groups using independent sample *t*-tests.

#### Functional Connectivity Analysis

**Figure 2:**
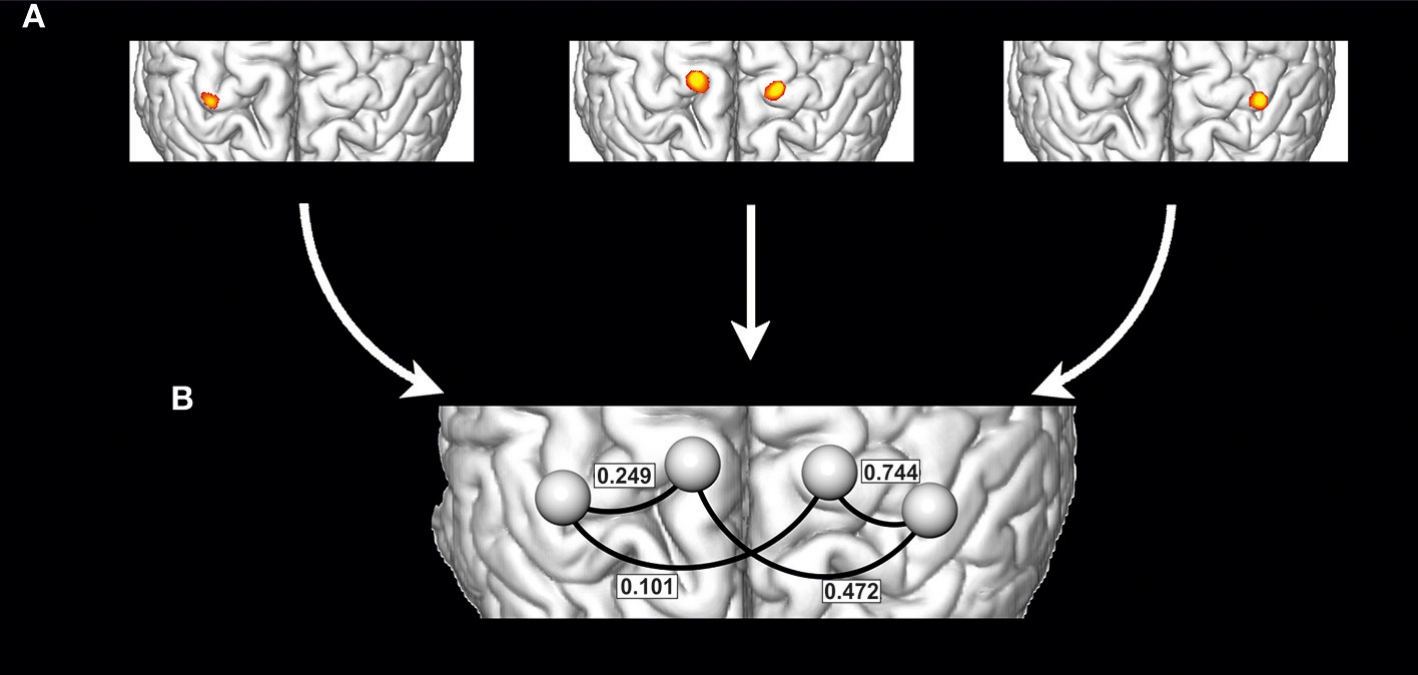
Schematic illustration of the generation of individualized seed ROIs within a single subject: **(A)** Definition of primary motor cortex activations based on the single subject task contrasts, which are activation of right arm (**A**, left), activation of left arm (**A**, right) and forced abdominal respiration (**A**, middle) (schematic drawing). For every activation the peak activity voxel was defined and 6 mm spherical ROIs were defined around that peak activation and then used to estimate task-based ROI-to-ROI connectivity networks separately for each task **(B)** (values from Table 2 and condition “moving diseased arm,” the right side corresponds to the diseased arm's hemisphere).

Preprocessing of imaging data and their statistical analysis was performed using SPM12 (Wellcome Trust Center for Neuroimaging, UK), the CONN functional connectivity toolbox (22) version 14 and in-house developed MATLAB (The Mathworks, Natick, MA, USA). All calculations were done with a minimum of model assumptions at the individual level using ROI-to-ROI analyses of individual seed regions derived from single-subject task activation data (23). This allows considering between patient variability concerning neuroanatomy, physiology, and pathophysiology and ensures that functional connectivity is calculated between truly active brain areas in every patient/subject (24). Data preprocessing for the individual functional connectivity analysis followed and was reduced to a minimum (25). Thus, preprocessing only involved slice time and motion correction followed by smoothing using a 5 mm full-width-at-half-maximum Gaussian filter. For the generation of individual patient-specific seed ROIs single-subject brain activation maps were calculated with SPM12 and 6 mm spherical ROIs were defined around the peak activation within the critical motor network areas. Based on our hypothesis these were the arm and diaphragm representations within the primary motor cortex (M1) of both hemispheres. Despite generally weak diaphragm activities, due to the high 7T contrast to noise ratios, individual definition of primary arm and diaphragm activity was unequivocally possible in all patients/subjects (see Figure 2 for a schematic illustration). Prior to the connectivity analysis confounding signals like residual motion or physiological artifacts were corrected using the aCompCor method (26). Corrected time-series were then used to estimate task-based ROI-to-ROI connectivity as primary outcome measure (22). To this end mean signal time courses were extracted within the four ROIs separately for each task and subject and used to calculate Pearson correlations between ROI pairs of interest. The resulting ROI-to-ROI estimates were finally converted using Fisher's Z-transformation and submitted to a 2 × 2 × 3 mixed-model repeated measures ANOVA. This full model of variance with the factors Group (two levels: controls, patients), Condition (three levels: diseased/left arm, healthy/right arm, forced inspiration) and Connection (four levels: diseased/left arm ROI to ipsilateral diaphragm ROI, diseased/left arm ROI to contralateral diaphragm ROI, healthy/right arm ROI to ipsilateral diaphragm ROI, healthy/right arm ROI to contralateral diaphragm ROI) allows to focus on the difference in functional connectivity between patients and controls, within one framework. All effects and contrasts were Greenhouse-Geisser corrected when sphericity was violated. Connectivity to the bihemispheric diaphragm ROI was calculated by combing the connectivity values of the individual arm ROI to the ipsi- and contralateral diaphragm ROI while conducting a specific movement. In addition, effect size calculations were performed (partial η^2^).

**Table 2:** Functional connectivity values (fisher's *z*-values) of the phrenic nerve patients separated per subject and condition.

	**Diseased arm ROI to the diaphragm ROI of the**						**Healthy arm ROI to the diaphragm ROI of the**					
	**Ipsilateral hemisphere**			**Contralateral hemisphere**			**Ipsilateral hemisphere**			**Contralateral hemisphere**		
	**Moving healthy arm**	**Moving diseased arm**	**Forced inspiration**	**Moving healthy arm**	**Moving diseased arm**	**Forced inspiration**	**Moving healthy arm**	**Moving diseased arm**	**Forced inspiration**	**Moving healthy arm**	**Moving diseased arm**	**Forced inspiration**
Pat. 1	0.310	0.903	−0.071	0.375	0.550	−0.127	0.158	−0.185	−0.376	−0.192	−0.403	−0.399
Pat. 2	0.196	0.405	0.147	0.182	0.310	0.264	0.300	0.609	0.230	0.026	0.288	0.094
Pat. 3	0.711	0.930	0.485	0.220	0.768	0.186	0.749	0.214	0.434	0.313	0.139	0.459
Pat. 4	0.181	0.728	0.831	0.464	0.397	0.781	0.475	0.194	0.156	−0.236	0.150	0.027
Pat. 5	0.343	0.557	0.759	0.794	0.236	0.802	0.493	0.365	0.621	0.000	0.263	0.567
Pat. 6	0.612	0.943	0.310	0.458	0.574	0.106	0.697	0.302	0.256	0.300	0.171	0.307
Mean (std)	0.392 (0.220)	0.744 (0.223)	0.410 (0.350)	0.415 (0.219)	0.472 (0.195)	0.335 (0.376)	0.478 (0.225)	0.249 (0.260)	0.220 (0.336)	0.035 (0.234)	0.101 (0.254)	0.392 (0.220)

*Last row represents the arithmetic mean of the individual values and their standard deviation in brackets*.

## Results

The clinical investigations mentioned above confirmed successful and independent innervation of bilateral diaphragm and unilateral biceps in all patients and no change of breathing patterns during arm movements. All participants could perform all fMRI tasks as requested and showed expected activations. Concerning head motion analysis, no significant difference (*df* = 16, *t* = −0.546, *p* = 0.593) was found for the mean frame-wise displacement between both groups (healthy control mean = 0.82 mm, *SD* = 0.296; patients mean = 0.92 mm, *SD* = 0.434). For the maximal displacement across the whole session again no significant difference occurred (*df* = 16, *t* = −0.499, *p* = 0.624) when comparing the two groups (healthy control max = 2.95 mm, *SD* = 1.14; patients max = 3.32 mm, *SD* = 2.07).

Individual task-based ROI-to-ROI analysis exhibited consistent connectivity changes for all three tasks (see Tables 2, 3). The 2 × 2 × 3 mixed-model repeated measures ANOVA revealed a significant main factor Connection (*df* = 1, *F* = 14.88, *p* < 0.000) as well as significant interactions between Connection × Group (*df* = 3, *F* = 4.654, *p* = 0.006) and Connection × Condition (*df* = 6, *F* = 5.870, *p* = 0.001 GG corrected). Next to these effects the 3-way interaction Connection × Condition × Group yielded significance (*df* = 6, *F* = 3.891, *p* = 0.002) indicating that patients differed from healthy subjects when comparing connectivity values of the left/diseased and right arm. Corresponding to our primary hypothesis, Table 2 shows a considerably increased arm—diaphragm connectivity for the diseased arm compared to the healthy arm. This is true for both diaphragm ROIs but with a dominance of the ipsilateral connection. The dominance of ipsilateral over contralateral connections was a consistent finding for both groups. Following our neuroplastic hypothesis we then performed one-sided univariate linear contrasts within the ANOVA model separately for patients and controls. For patients the connectivity of the arm ROI (moving arm) to the bihemispheric diaphragm ROI was compared between the diseased and the healthy arm. For controls the connectivity of the arm ROI to the bihemispheric diaphragm ROI was compared between the non-dominant (left) and the dominant (right) arm. With patients we found a significant connectivity increase for the diseased arm (*df* = 1, *F* = 15.668, *p* = 0.011, partial η^2^ = 0.758). With healthy subjects no connectivity difference was found between arms (*df* = 1, *F* = 0.537, *p* = 0.479, partial η^2^ = 0.047).

**Table 3:** Functional connectivity values (fisher's *z*-values) of the healthy controls separated per subject and condition.

	**Left arm ROI to the diaphragm ROI of the**						**Right arm ROI to the diaphragm ROI of the**					
	**Ipsilateral hemisphere**			**Contralateral hemisphere**			**Ipsilateral hemisphere**			**Contralateral hemisphere**		
	**Moving right arm**	**Moving left arm**	**Forced inspiration**	**Moving right arm**	**Moving left arm**	**Forced inspiration**	**Moving right arm**	**Moving left arm**	**Forced inspiration**	**Moving right arm**	**Moving left arm**	**Forced inspiration**
Cont. 1	0.155	0.226	0.484	−0.094	0.293	0.437	0.341	0.166	0.341	−0.262	0.147	0.446
Cont. 2	0.247	0.599	0.544	−0.194	0.262	0.342	0.133	−0.042	0.135	0.105	0.240	0.147
Cont. 3	1.267	0.251	0.388	0.281	−0.130	0.146	0.526	0.498	0.542	0.464	0.406	0.567
Cont. 4	0.953	0.784	0.523	0.054	0.348	0.368	0.310	0.559	0.433	0.129	0.421	0.876
Cont. 5	0.341	0.368	0.499	−0.046	0.241	0.264	0.384	0.424	0.402	0.384	0.418	0.523
Cont. 6	0.982	0.751	0.760	0.097	0.727	0.479	0.870	0.159	0.106	0.259	0.268	0.136
Cont. 7	0.645	−0.031	0.191	0.214	0.243	0.116	0.787	0.335	0.444	0.198	0.232	0.244
Cont. 8	0.555	0.872	0.618	0.177	0.541	0.418	0.181	0.385	0.449	0.168	0.471	0.525
Cont. 9	0.604	0.390	0.502	0.106	0.252	0.393	−0.057	0.378	0.252	−0.206	0.329	0.240
Cont. 10	1.017	0.357	0.507	0.085	−0.093	0.399	1.189	0.924	0.749	−0.059	0.243	0.498
Cont. 11	0.356	0.533	0.035	−0.046	−0.110	0.048	0.501	0.281	0.164	−0.317	−0.065	0.187
Cont. 12	0.611	0.508	0.299	0.004	0.060	−0.017	0.477	0.169	−0.135	0.014	0.383	0.221
Mean (std)	0.644 (0.346)	0.467 (0.260)	0.445 (0.193)	0.053 (0.135)	0.219 (0.259)	0.282 (0.167)	0.469 (0.344)	0.352 (0.245)	0.323 (0.233)	0.073 (0.247)	0.291 (0.149)	0.384 (0.224)

*Last row represents the arithmetic mean of the individual values and their standard deviation in brackets*.

Since several studies [see Bartlett and Leiter (27) for a review] have reported a basic coupling between arm and diaphragm areas cross various tasks, we also tested connectivity changes during forced inspiration. In detail, patients' connectivity of the arm ROI to the bihemispheric diaphragm ROI was tested during forced inspiration, again comparing connectivity values for the diseased and the healthy arm. In contrast to arm movements, forced inspiration did not generate a significant connectivity increase between the diseased arm and bihemispheric diaphragm (*df* = 1, *F* = 1.971, *p* = 0.219, partial η^2^ = 0.283) in the patient group. The results indicate a highly specific connectivity increase required to move the diseased arm and exclude a global connectivity change or effects due to simultaneously occurring task-related respiration.

Observations obtained within groups were further confirmed when comparing between groups (patients vs. healthy controls). The patients' diseased arm ROIs connected stronger to the ipsilateral diaphragm ROIs when compared with the weaker (non-dominant) left arm of healthy subjects (*df* = 1, *F* = 4.924, *p* = 0.041, partial η^2^ = 0.235).

## Discussion

In this study we describe a new rehabilitative mechanism of primary motor cortex after peripheral trauma. Although a large body of evidence describing the flexibility of the human cortex to reorganize already exists, only a few functional imaging studies describe cortical reorganization in response to peripheral nervous system damage (9). Here the driving factor for neuroplasticity is the changed information flow between the somatic periphery and the brain but not a brain lesion. In this context, recently a new type of neuroplasticity has been described: a change of a monofunctional to a bifunctional area where the brain area sustains its original function and adds a new one (15). Here, we used functional ROI-to-ROI connectivity analyses to investigate the mechanisms behind this neuroplastic effect. Our main finding shows that the phrenic nerve patient group exhibits a consistent increase in connectivity between the area of the diseased arm and the bilateral diaphragm areas while moving the diseased arm. Most likely, the origin of this increased functional connectivity is mediated via horizontal cortical interconnectivity (28, 29). Both, within-subject and between-subject comparisons showed a significant increase of the diseased arms connectivity to the diaphragm areas. These connectivity changes allow the perpetuation of the original function of the completely denervated arm cortex (which was found to remain highly active during previous brain activation studies) and our data indicate the following rehabilitative mechanism: the denervated arm area generates “its” arm movements via connection to multifunctionally transformed diaphragm cortex, which then activates the biceps muscle. Evidently, both diaphragm areas work together to support this rerouting effect. The effect is highly specific for diseased arm movements since no other task (e.g., breathing) generates a comparable connectivity increase. It is noteworthy, however, that our healthy subject data also show a kind of basic default coupling between the arm and the diaphragm areas during arm movements. This coupling has already been described, in particular in musicians playing the piano but also cross various other tasks (27, 30). In our patient data this basic motor-breathing coupling cannot explain our diseased arm findings since we found no arm connectivity change during forced respiration and no change of clinical breathing patterns during arm movements (9). Hence, the original breathing function of the diaphragm area is accomplished independently from the new arm movement function. This is a critical point given that the diaphragm area has to transform into a bifunctional brain area supporting abdominal respiration as well as arm movement.

Concerning our patient population, brachial plexus lesions are commonly treated using an end-to-end technique. The patients of this study, however, were operated using an end-to-side coaptation. While the first option sacrifices a complete nerve function like that of an intercostal nerve, the here used approach preserves the nerves' original function, by connecting the denervated ending of the musculocutaneous nerve to the side of a fully functional phrenic nerve via double interpolations (31). For both clinical approaches functional plasticity of the central nervous system areas have to follow different routes. Two previous studies found global network changes and bilateral brain activation changes in different brachial plexus patients. Investigating pan–brachial plexus injury patients pre- and post-end-to-end operation, showed changes within the sensorimotor network and higher cognitive networks like the salience network or the default-mode network (32). Comparing patients that improved following the operation with non-improvers, connectivity within the left hemispheric sensorimotor network served as a particularly good predictor for recovery. Another group of patients is one suffering from brachial plexus birth injury but without any surgical interventions. Here a shoulder and elbow movement task was used to investigate activation changes related to cerebral reorganization (33). The authors observed increased activation solely within primary sensorimotor cortex related to the diseased side but bilateral activation increases in secondary sensorimotor areas. This bilateral neuroplastic component seems to be important for reconstitution of function since it was also found in our new connectivity results.

A limitation of our study certainly concerns the small number of end-to-side phrenic nerve patients existing worldwide. Whenever clinically possible, standard procedures are preferred for surgery. Our study includes about 25% of existing patients. It is important to realize that these patients represent a unique pathophysiological model, and the new type of intracortical connectivity changes could not have been detected with other patient groups. Note also, that the connectivity changes described were found in every single patient which renders it likely that the results are representative for this peripheral reconstruction type. Since interindividual functional variability in patients may be large and might complicate clinical group analyses, we focused on individual patient results (24) and the influence of experimental or post-processing related noise was minimized using ultrahigh field data with improved signal to noise ratio (18) and an analysis which excludes brain normalization steps.

## Data Availability Statement

The datasets generated for this study are available on request to the corresponding author.

## Ethics Statement

The studies involving human participants were reviewed and approved by Ethikkommission der Medizinischen Universität, Wien Borschkegasse 8b/E06, 1090, Wien. The patients/participants provided their written informed consent to participate in this study.

## Author Contributions

FF, RB, EM, and RS contributed to the study conception and design. FF, AA, RR, and EM contributed to the data acquisition. FF and RB contributed to the data analysis, drafting of the manuscript, and preparing the figure.

### Conflict of Interest

The authors declare that the research was conducted in the absence of any commercial or financial relationships that could be construed as a potential conflict of interest.
